# The Maritime Medical News

**Published:** 1888-12

**Authors:** 


THE MARITIME MEDICAL NEWS.—This is the title of a new medical journal to be published bi-monthly at Halifax, N. S., that made its first appearance in November, 1888. Hitherto, as the new journal announces editorially, there has been no medical publication in the Maritime Provinces, which would appear a sufficient reason for the establishment of this new candidate for professional favor. We extend a cordial greeting to our ably-edited and well-printed contemporary.

---

DR. CHARLES H. MERZ, the house physician to University Hospital at Cleveland, Ohio, April 25, 1887, said: "I have made use of Papine for some time past, both in hospital and private practice, and find it a most agreeable substitute for morphine and opium. It is the anodyne *par excellence*."

---

THE CANADA MEDICAL RECORD commenced a new volume with its October number, when it appeared in a fresh and beautiful dress. Our esteemed contemporary merits the confidence of its constituency, and is a credit to the journalistic enterprise of the Canadian metropolis.

